# Modeling spatiotemporal patterns of microplastic pollution in the lupit river using multilinear regression

**DOI:** 10.1038/s41598-025-23619-2

**Published:** 2025-10-31

**Authors:** Katharina Raab, Ralf Wagner, Marie Therese Sales, Christer John Uy, Benedict Salazar

**Affiliations:** 1 School of Economics and Management, University of Kassel, Kassel, Germany; 2 Chemical Engineering Department, University of St. La Salle, Bacolod City, Philippines

**Keywords:** Water quality, Weathering, Pollution sources, Lupit river, Microplastics, Ecology, Ecology, Environmental sciences

## Abstract

Despite increasing awareness of microplastics as contaminants, their sources and abundance factors remain poorly understood. This study found widespread microplastic pollution in the Lupit River. Microplastic concentrations were determined from surface water samples once per season. Samples were analyzed across rural, residential, informal settlement, and commercial zones during both seasons. Microplastic concentration (particles/m^2^) was modeled using multiple linear regression with four predictors: population, seasonality, macroplastic frequency (items/m-hr), and volumetric flow rate (m^3^/s). To enhance model stability, multicollinear variables (velocity, width, and depth) were removed. Concentrations were lower in the wet season due to dilution and flushing and higher in the dry season likely from accumulation and weathering. The final model showed strong explanatory power (R^2^ = 0.690, adjusted R^2^ = 0.643), with population and seasonality as significant predictors (*p* < .001). Seasonality had a negative effect (β = – 621.90) due to dilution. Surprisingly, population correlated negatively (β = -0.0217), suggesting better waste infrastructure in denser areas. Macroplastic abundance and flow rate were not statistically significant due to slow weathering and local microplastic accumulation. The model’s standard error (301.6 particles/m^2^) accounted for 16.9% of the mean concentration (1,780 particles/m^2^), signifying good prediction accuracy. The developed predictive model provides a low-cost tool for estimating microplastic levels in data-scarce areas which can assist agencies in tracking pollution trends over time and to assess the effectiveness of waste management policies. Future research should validate the model across different catchments and temporal scales to enhance its utility in long-term monitoring.

## Introduction

The growing threat of plastic pollution in marine waters now extends to freshwater systems, which show increased evidence of contamination by microplastics. The expanding problem of river pollution needs community involvement in order to support widespread data collection and encourage local action to mitigate plastic pollution. Freshwater ecosystems, encompassing rivers, streams, lakes, and ponds, are more highly susceptible to microplastic pollution due to their function as major repositories of terrestrial runoff, which frequently carries contaminants due to inadequate waste management practices and compromised landfill integrity^1^. This expanding environmental problem threatens rivers, which play a vital role in conserving biodiversity and supporting human activity, serving as essential life support systems for both people and the environment while supplying livelihoods through their integration with human activities.

Plastics are engineered for durability and chemical resistance, resulting in slow weathering rates in natural environments. Simulated studies have been conducted to determine long-term microplastic weathering under various aqueous conditions. Polypropylene was the first polymer to fragment, after nine months, whereas polyethylene showed minimal weathering even after 18 months in freshwater, brackish water, and seawater, highlighting the extended persistence of these materials in aquatic ecosystems^2^. Macroplastics are larger plastic debris (greater than 5 mm) and are typically visible and less degraded, whereas microplastics comprise plastic particles smaller than 5 mm that often result from macroplastic breakdown^3^. Their shapes include microbeads, fibers, fragments, film, foam pellets, and filaments^4^, and they may be either primary or secondary microplastics. Primary microplastics are manufactured by cosmetics industries, whereas secondary microplastics occur due to plastics’ weathering in the environment^5^. Color is the key feature in visually identifying microplastics, with brightly colored particles more likely to be recognized as plastic^6^. These particles’ tiny dimensions enable them to penetrate the aquatic food web, where they are ultimately ingested by marine animals and become vectors for pollutant and microorganism transfer.

The potential hazards of microplastics in water arise from their ability to carry chemical pollutants and microbiological pathogens. To date, no comprehensive studies have examined the full range of effects that microplastic ingestion may have on human health. However, research has shown that plastics can carry loosely bound chemicals, including additives and monomers, which are toxic to living organisms. Emerging evidence indicates that microplastics can accumulate in specific human tissues and organs, such as the placenta and lungs, potentially influencing immune system function and contributing to other health concerns^7^. Microplastics can break down under environmental conditions, forming particles small enough to be ingested. While present in aquatic environments, they often develop thin biofilms of microorganisms on their surfaces, which may ultimately enter drinking water systems. This is particularly concerning in regions where water bodies also serve as outlets for untreated sewage, increasing the risk of undetected fecal contamination associated with microplastics. Thus, microplastics in water pose risks related to both chemical and microbiological contamination.

In tropical river systems, relatively few studies have empirically quantified both spatial and temporal variation, and associated these patterns to source contributions. The study of^8^ in the Langat River, Malaysia, showed that microplastic abundance varied strongly with both discharge (temporal variation) and land use (spatial variation). This study was conducted through a year-round sampling across forested, residential, industrial and urban sites. Another study in the Perak River, Malaysia, investigated the presence and characteristics of microplastics in river water used for drinking water supply. Results of the study showed that severe microplastic contamination exists in the Perak River for both the wet and dry seasons^9^).

Despite the growing recognition of microplastics as an emerging contaminant, accurately determining their sources and the factors contributing to their abundance in freshwater ecosystems remains a complex undertaking^10^). There remains a limited understanding of the key factors influencing microplastic abundance across different land-use zones and seasons in freshwater systems. This study addresses these gaps by providing comprehensive empirical data on microplastic concentrations in the Lupit river across rural, residential, informal settlement and commercial zones during both wet and dry seasons.

**Fig. 1:**
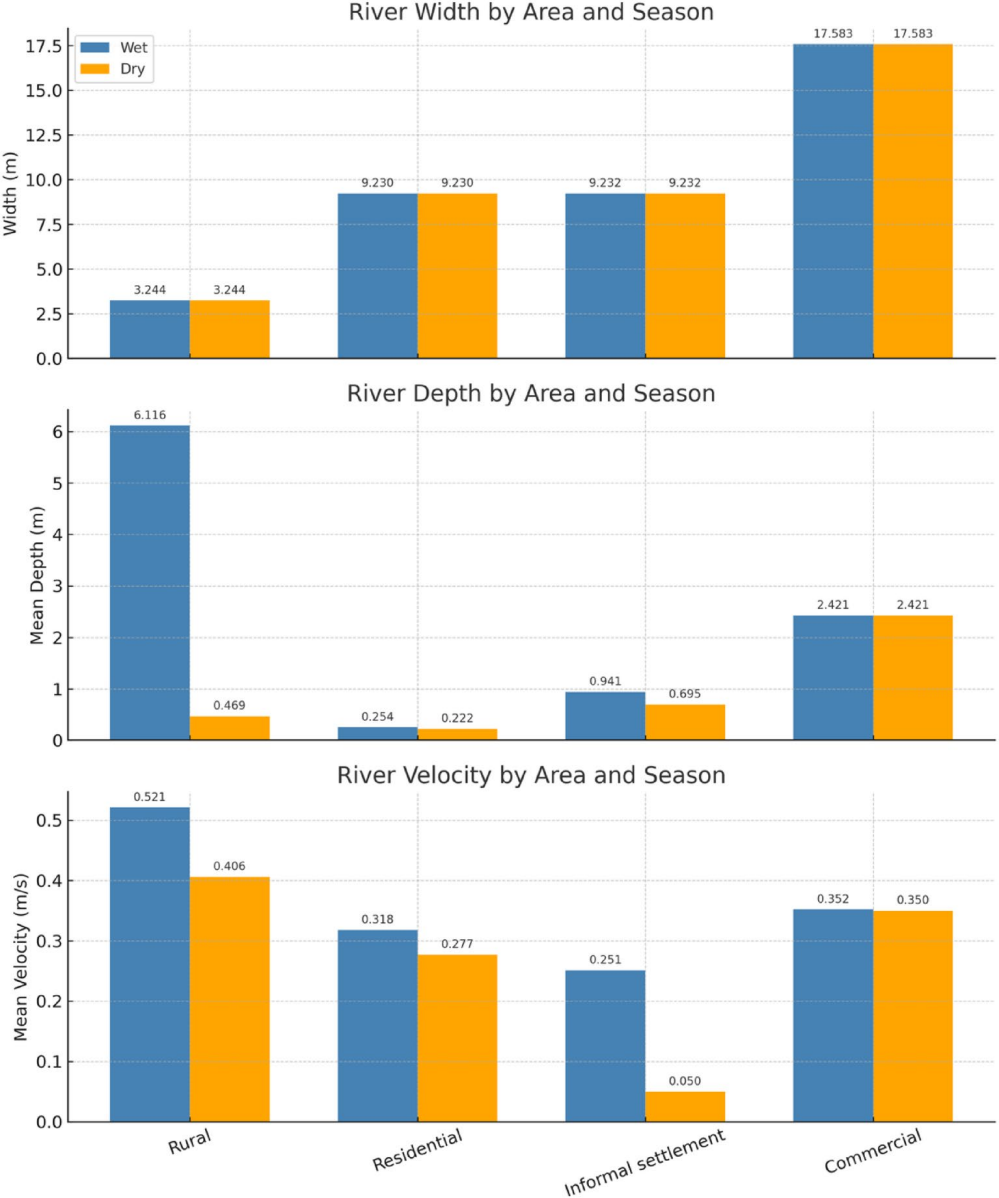
Characteristics of the river during the wet and dry seasons.

Current studies have identified some predictors of microplastic pollution in freshwater systems that include their transport and fate of dynamics, vertical distribution in river water columns, and interactions with aquatic vegetation. These factors influence not only the movement but also the retention and accumulation of microplastics in inland water. Insights from recent reviews^11–13^ highlight the importance of incorporating such parameters when assessing microplastic contamination levels. This difficulty stems from the diverse and often diffuse pathways by which these particles enter river systems. Recent research increasingly highlights the importance of both spatial and temporal factors in governing the distribution of microplastics^14^. For example, studies have linked microplastic concentrations to spatial factors like population density, land use, and sediment characteristics, as well as temporal factors such as seasonal variations, precipitations, and water flow^15^^16^;. However, many studies focus on a limited set of these variables or analyze them independently. There is a clear need for research that integrates these key drivers – including both anthropogenic (e.g., population, and microplastic presence) and hydrological dynamics (e.g., volumetric flow rate and seasonal cycles) – into a single predictive framework to better understand their combined influence on microplastic abundance. The multiple linear regression model in this study successfully identifies the significant influence of anthropogenic and large-scale hydrological factors. However, it is a simplification of a complex system and does not account for the intricate relationship between microplastic properties (e.g., density and shape)^17^ and the fine-scale hydrodynamic processes that govern their transport and vertical distribution within the water column. A more comprehensive predictive model would ideally integrate these factors which could be a focus for future research (Fig. 1).

**Fig. 2:**
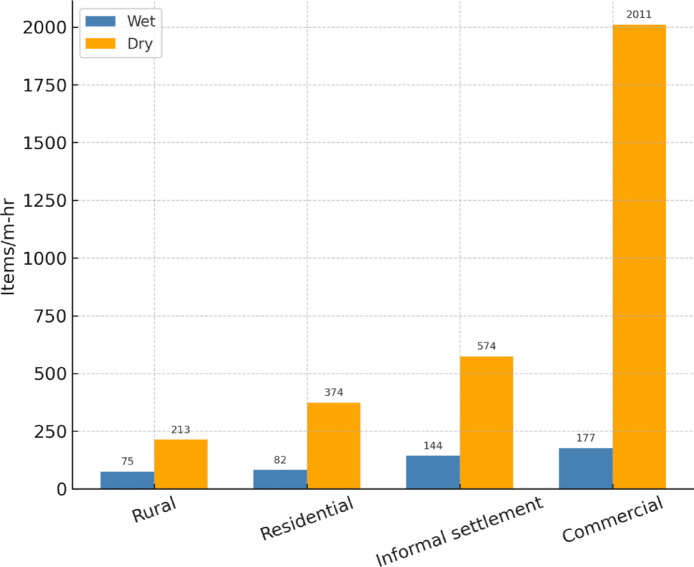
Macroplastic analysis for the Lupit River.

The Lupit River in Bacolod City, Philippines, provides a compelling case study to address this research gap. As one of five major river systems in a city with a rapidly growing population, the Lupit River is a critical waterway that is highly susceptible to plastic pollution. The river is bounded by six barangays, which contain nearly 30% of the city’s total population, and a significant portion of its banks are home to informal settlements. The river’s high levels of visible solid plastic waste (as shown in Fig. 2) strongly suggest that macroplastic debris is a major source of secondary microplastics.

Based on these observations, this study assumes that the spatial and temporal variations in microplastic concentration in the Lupit River are primarily determined by a mix of anthropogenic stresses (population and microplastic frequency), hydrological parameters (river width, depth, and volumetric flow rate), and seasonal cycles (dry and wet seasons). Currently, there is no previous study that has used this specific combination of predictors to model microplastic concentrations in a developing-country urban river using multiple linear regression approaches. This research, therefore, offers a novel contribution by using an integrated approach of investigating how various factors, such as population, seasonality, anthropogenic activities, and the river’s characteristics, influence microplastic pollution trends in the Lupit River.

A predictive equation was developed to connect these variables through a well-fitting statistical model, offering a practical tool to inform targeted management and mitigation strategies aimed at conserving water quality and ecosystem health.

## Results

Figure 1 presents the river’s characteristics—width, mean depth, and mean velocity—during the wet and dry seasons. In the wet season, the commercial area exhibited the greatest depth and width, as it lies only a few kilometers from the estuary. In contrast, the rural section had the narrowest width due to dense vegetation along its embankments. Hotspot identification was guided by the criteria used in^18,19^, which included visible solid waste leakage, lack of formal waste collection, proximity to the river system, and the presence of illegal dumpsites. These hotspots were selected as key sampling areas because of their high potential to contribute to microplastic pollution. The shallowest river depth was observed in the residential sector, likely due to upstream quarrying activity near the hotspot. The rural sector had the highest flow velocity, attributed to increased upstream water levels during the wet season and the steeper gradient of that river section. In contrast, the lowest mean velocity was found in the informal settlement area, where large rocks obstructed the flow of water.

Dry season data were collected under ambient temperatures of approximately 30 °C. Compared to the wet season, the river’s water level dropped by an average of 21%, primarily due to seasonal variation. A strong El Niño event, which began in January 2024 and persisted through February, further exacerbated the decline in water levels^20^. As downstream river flow is dependent on upland water sources, the prolonged lack of rainfall led to significant reductions in flow. Specifically, the Lupit River experienced an average 29% decrease in flow velocity, which contributed to a 70% reduction in volumetric flow rate—an effect attributed to the El Niño phenomenon.

**Fig. 3:**
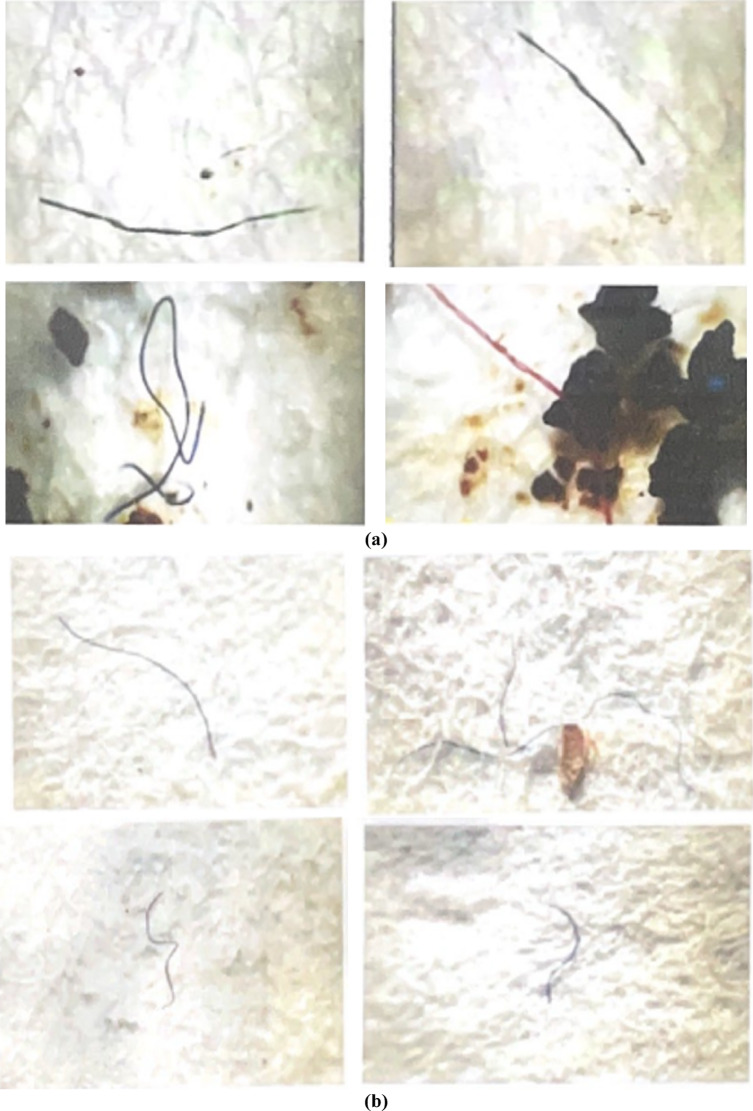
Microscopic analysis of microplastics in the Lupit River during the (**a**) wet season and (**b**) dry season.

Direct dumping of plastic and other waste materials into rivers is a prominent anthropogenic activity that significantly contributes to microplastic pollution in freshwater system^21^. This microplastic pollution can be attributed to the macroplastic pollution present in the river water. Figure 3 shows the results for macroplastic count in the Lupit River.

The spatial pattern of macroplastic pollution in the Lupit River indicates an evident downstream increase in plastic loading, suggesting that land-use categories and anthropogenic activities contribute more strongly to pollution levels than population size alone. The rural area had the largest population (73,947), and the upstream site had the fewest macroplastic items during both the wet (75 items/m-hr) and dry (213 items/m-hr) seasons. Much greater plastic loads were recorded downstream in the commercial and informal settlement areas. The commercial area, which is situated close to the estuary and had a population of only 33,372, had the greatest macroplastic accumulation, with 177 items/m-hr during the wet season and 2,011 items/m-hr in the dry season. The observed increase in macroplastic pollution from the upstream rural area to the downstream commercial zone of the Lupit River can also be attributed to the river’s characteristics. Upstream areas have narrower channels and shallower depths, which can promote the flow of plastics downstream. The downstream areas often exhibit wider channels and greater depths, allowing the settling and accumulation of macroplastics. Slower water allows denser objects to sink and remain in place, especially during periods of low discharge. Furthermore, because it lies near the sea, the segment of the river in the commercial area was influenced by tidal activity. At high tide, incoming water can retard downstream movement, causing macroplastic waste to remain stuck in the commercial zone.

The determination of the presence of microplastics in the samples was based on particle size, shape, and color. The optical microscopy results showed the presence of microplastics in the Lupit River as shown in Fig. 3. Fiber-like particles are visible, and the color is predominantly brighter than the background color. Microplastics were present in all sectors of the Lupit River.

**Fig. 4:**
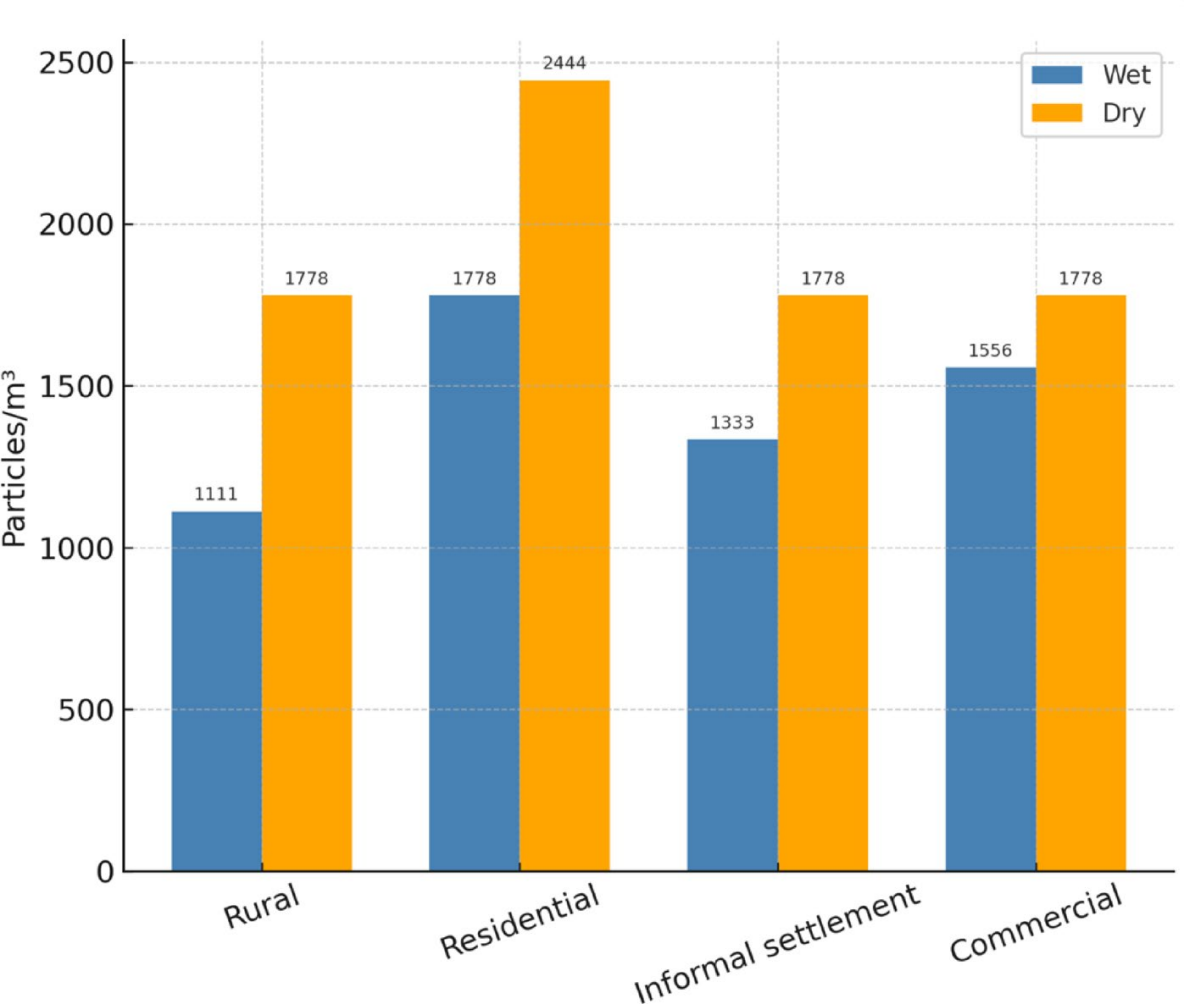
Microplastic analysis of the Lupit River.

Figure 4 shows that microplastic levels during the wet season were lower than in the dry season, suggesting that during the rainy season, some plastics in the water system may be transported away and not degrade at the study site. Conversely, during the dry season, low water levels and reduced flow exposed plastics trapped in boulders to weathering, leading to eventual fragmentation into smaller particles.

In the wet season, the rural area exhibited the lowest MPA, which was attributed to the economic conditions of the populace, as this is a mainly agricultural area. The limited purchasing power of farmers reduced the consumption of plastic-packaged instant food items. Conversely, the residential area showed the highest MPA during the wet and dry seasons, primarily due to bulky plastic bottles and disposable diapers becoming lodged in boulders for extended periods. Moreover, among the areas, the residential sector had the lowest mean depth for both seasons, which can affect the accumulation of microplastics. Shallow areas in the river have more turbulence, which can keep the microplastics suspended in the water column as opposed to deep meandering rivers, which have low turbulence. The laminar flow of deep rivers can allow the microplastics to settle on the riverbed over time. Since the sampling was done on the surface of the river water, there is no definitive evidence to suggest that the riverbed may have a higher MPA, as it was not included in the study.

The Lupit River is among the drinking water sources of Bacolod City. At present, water treatment processes for surface water do not include the removal of microplastics, and the Philippine Standards for Drinking Water and DAO 2016-08 (Water Quality Guidelines) do not set standards for microplastics in surface water. Moreover, there is no worldwide standard measure of microplastic abundance in surface water, so not all published studies can be compared directly. Consequently, only studies quantifying microplastics in the same units (particle/m^3^) for surface water were selected for comparison. These comparisons may be inaccurate owing to size differences in particles, sampling techniques, and the timing of sampling, but all these studies provide a general description of the global trends in microplastic pollution.

Osorio et al.^22^ reported microplastic concentrations ranging from 1580 to 57,665 particles/m^2^ in five Philippine rivers (Cañas, Pasig, Parañaque, Tullahan, and Meycauayan). Other rivers, such as the Lawaye River (3,333 particles/m^2^^23^), the Tunasan River (7547 particles/m^2^^24^), and Makati Creek (833 particles/m^2^^25^), also showed significant levels of microplastic pollution. The results from the Lupit River are consistent with these findings, indicating that microplastic contamination is a widespread environmental problem in the region. This trend suggests that Philippine river systems are heavily affected by extensive plastic waste mismanagement, excessive plastic consumption, and insufficient waste collection and recycling infrastructure. Consequently, plastic pollution enters rivers through surface runoff, improper disposal practices, and inadequate treatment facilities.

## Discussion

A four-variable multiple linear regression model was constructed to examine the anthropogenic and environmental predictors of microplastic pollution in the Lupit River. A key limitation of the study is that the characterization of microplastics did not extend to morphological or polymer-type analysis, due to resource constraints. This omission restricts the ability to trace microplastics back to specific sources and limits comparability with studies that include polymer identification. Consequently, while the study provides robust quantitative insights into microplastic abundance, it cannot fully address questions of origin, degradation pathways, or material-specific ecological impacts. The analysis was based on a total of 36 data sets collected across 2 distinct seasons (wet & dry). The samples were stratified by sectors to capture a representative range of environmental conditions. For the Rural, Residential, and Informal settlers, 5 data sets per sector per season were collected. Conversely, only 3 data sets per season were collected for the commercial sector. The smaller sample size for this specific sector was due to unforeseen logistical issues, namely, accessibility concerns caused by an increase in the tide level during sampling, as the sites were located near a coastal area. Multiple regression modeling does not require perfectly equal sample sizes across all groups or predictors. Instead, the primary requirements for a valid analysis are that each predictor variable exhibits sufficient variance and that the underlying statistical assumptions are met. During the model fitting process, there was a necessity to exclude 5 data sets that were determined to be not statistically significant. This step is to improve the overall fit and predictive power of the model, thus having 31 data sets used for the modelling. Moreover, the variables of velocity, width, and depth, previously considered because of their possible hydrological effect, were omitted from the final model because they had high VIFs (> 10), strong multicollinearity, and were not statistically significant in initial runs. Table 1 shows the regression statistics of the final model.

**Table 1:** Regression statistics for the final model.

Regression Statistics	
Multiple *R*	0.830806017
*R*-squared	0.690238638
Adjusted *R*-squared	0.642583044
Standard error	301.6279029
Observations	31

The final model shows good explanatory power, with a multiple *R* of 0.831, reflecting a very strong correlation between predicted and observed microplastic concentration. The coefficient of determination (*R*^2^) is 0.69, meaning that 69% of the microplastic concentration variance is explained by the included predictors. The adjusted *R*^2^ is also good at 0.643, indicating the robustness of the model after considering the predictors. The standard error of 301.6 particles/m^3^ shows moderate predictive accuracy. This represents 16.9% of the average microplastic concentration of 1,780 particles/m^3^. This may be due to unmeasured environmental or anthropogenic factors. The model’s statistical significance was established through ANOVA (*F* = 14.48, *p <*0.000001), which illustrates that the predictors collectively account for a very large percentage of the variation in microplastic concentration.

The population variable was statistically significantly and negatively correlated with microplastic concentration (β = −0.0217, *p <* .001). The negative correlation is contrary to initial assumptions but consistent with a previous study in a comparable urbanizing watershed, where denser areas tend to have more developed waste collection systems and better plastic capture^26^. This insight is particularly relevant in a developing-world context such as Bacolod City. While other studies have focused on larger-scale models, Hyper-local data suggests a tangible reality: denser, more formal commercial and residential sectors may have better waste capture, whereas less-populated, peri-urban, or informal settlement areas may contribute disproportionately more unmanaged waste per capita. This finding challenges the simplistic use of population as a direct proxy for pollution and instead points to infrastructure disparity as the primary driver.

Seasonality was also an important predictor (β = −621.90, *p <* .001), as the wet season was related to significantly lower concentrations of microplastics than the dry season. These findings align with^8^ which similarly observed higher microplastic concentrations during dry seasons due to reduced dilution and increased pollutant retention. This outcome is in accord with other work emphasizing the dilution or flushing of microplastics downstream or into sediment beds through rainfall and enhanced discharge^27,28^. The large coefficient highlights the magnitude of this effect in a tropical, monsoon-driven river system, where seasonal flushing can obscure underlying pollution loads. This demonstrates a key limitation of short-term or single-season monitoring campaigns, which risk underestimating the true extent of microplastic reservoirs that become more visible during the dry season.

Conversely, macroplastic count and volumetric flow rate had no statistically significant effects on microplastic concentration in the present model. The coefficient for macroplastic count was practically zero (β = −0.00016, *p* = .0.999), implying that major visible plastic litter is not likely to reflect directly on the amount of microplastics over the time of sampling. The macroplastics number is not statistically correlated with microplastic density, because microplastics usually emerge from long-term weathering of plastics through processes such as UV exposure, mechanical wear, and chemical weathering^2^. As a result, newly deposited macroplastics do not necessarily affect microplastic density right away, since fragmentation into microplastics happens progressively over time, usually outside the short-term limit of sampling or observation. Volumetric flow rate was also found to have no significant effect (β = −2.08, *p* = .678), which may suggest that microplastics are more controlled by localized retention and human contribution than by the volume of water flow itself. The visible macroplastic waste and invisible microplastic pollution are decoupled phenomena. Cleanup efforts targeting microplastics serve as preventive measures for future microplastic load but do not address existing contamination driven by historical plastic inputs.

Although the coefficients of the final model are calibrated to the Lupit River, the broader insights are transferable to other urbanizing watersheds, particularly in tropical regions with pronounced wet and dry seasons (e.g., Southeast Asia, Latin America, South Asia). The results demonstrate that infrastructure disparity, seasonal flushing, and the legacy nature of plastic degradation are the dominant drivers of microplastic pollution. These findings provide a conceptual framework that can guide the design of monitoring programs and management interventions in similar river systems globally.

To quantify the drivers of microplastic pollution in the Lupit River, a multiple linear regression model was constructed as shown in Eq. (1). This model incorporates the most significant environmental (e.g., volumetric flow rate, seasonal variability) and anthropogenic (e.g., population, macroplastic count) variables for the estimation of microplastic concentration. The derived predictive equation is a quantitative means of examining the contribution of various factors to microplastic levels in different sectors of the river.

$${\text{MPA = 3113}}{\text{.08 - 0}}{\text{.0217 (Population) - 621}}{\text{.90 (Wet season) - 0}}{\mathrm{.00016}}$$

1 $${\text{(Macroplastic count) - 2}}{\text{.08 (Volumetric flow rate)}}$$

The prediction estimate expression displays the coefficients associated with each predictor variable and the extent to which the MPA changes with a change in the individual predictor variable. The intercept is given at 3,113.08, which represents the microplastic concentration when all other predictor variables are zero.

The predictor factor for population is less than 1, which is not very strong, meaning it cannot influence the MPA. This indicates that for every one-person increase in population within the river sector, the predicted microplastic concentration decreases by approximately 0.0217 particles/m^3^, assuming all other variables are constant. The negative and statistically significant coefficient could imply that sectors with more population may have better waste management systems, infrastructure, or public awareness efforts that help reduce microplastic pollution. By contrast, less populated areas may lack such systems, leading to more unmanaged waste leakage. The results point toward the overriding effect of human population and seasonality on microplastic levels in tropical urban river systems such as the Lupit River. Although population is generally expected to add pollutant burdens, the negative correlation in this study could suggest infrastructure imbalances, whereby more rural or informal communities without systems for solid wastes add more microplastics per capita than larger, more developed sectors. This supports growing evidence that inadequate waste infrastructure, more than population size, drives plastic pollution in river systems^29^.

The wet season factor in the regression model, with a coefficient of − 621.90, indicates that the predicted microplastic concentration during the wet season decreases by approximately 622 particles/m^3^, assuming all other variables are constant. The possible confounding factors that may have affected this predictor are changes in human activity and improved waste management during the wet season. Most people lessen their outdoor activity during the rainy season, which could lead to less plastic product consumption. Moreover, the negative coefficient implies that increased water flow during the rainy season can dilute and flush the microplastics downstream, leading to a decrease in their concentration. The substantial decrease in microplastics during the wet season is consistent with regional studies that show flushing effects during periods of high rainfall, which can transport microplastics downstream or flush them into estuarine and marine systems. This seasonal phenomenon highlights the importance of time-stratified monitoring approaches that consider variability in rainfall and hydrological regimes.

The macroplastic count variable in the regression model has a very small coefficient of –0.00016 and is not statistically significant. This indicates that changes in the number of macroplastic items have no effect on the predicted microplastic concentration in the current model, meaning that macroplastics do not immediately contribute to microplastic levels, as their fragmentation takes time. Thus, short-term measurements of floating macroplastics may not reflect the microplastic content in the river at the time of sampling. The absence of strong correlation between macroplastics and microplastics in this study indicates that the presence of macroplastics may not be a good indicator of microplastic contamination in real time, as weathering to microplastics is a time-delayed, nonlinear process that depends on environmental exposure and physical stressors^30^.

The model’s volumetric flow rate variable has a coefficient of − 2.08, indicating that for every 1 m^3^/s increase in river flow rate, the predicted microplastic concentration decreases by about 2.08 particles/m^3^, assuming other variables are constant. The weak relationship between volumetric flow rate and microplastic concentration may be due to the dilution of microplastic concentration during an increase in water volume. It is possible that volumetric flow rate influences how microplastics are transported but does not directly affect their concentration at specific sampling points.

Furthermore, the omission of velocity, width, and depth from the final model further supports the notion that microplastic pollution is more strongly associated with terrestrial human processes and surface phenomena than with simple physical river characteristics, at least at the scale examined here.

## Conclusion

This study provides one of the first comprehensive spatiotemporal assessments of microplastic contamination in the Lupit River, offering both descriptive insights and a predictive framework. Its principal contribution lies in the construction of a regression model (R^2^ = 0.690) that isolates the dominant drivers of microplastic pollution in a tropical urban river system. Unlike previous studies that relied primarily on descriptive patterns, this work demonstrates the added value of a quantitative, predictive approach that can inform local policy and management.

The key findings show that population density and seasonality are the most significant predictors of microplastic concentration, whereas macroplastic counts and flow rate were not. This challenges the common assumption that visible macroplastic abundance translates directly into microplastic load and supports a growing body of literature emphasizing the legacy nature of plastic degradation^26,30^. The observed dry-season peak in concentrations reflects both hydrological and climatic factors, including reduced flow and enhanced weathering processes, consistent with global studies^27,28^. Residential zones emerged as persistent hotspots, underscoring the role of waste management infrastructure and local geomorphology in shaping exposure risk.

Despite the model’s explanatory strength, limitations must be acknowledged. The analysis was confined to a single river system, and while the predictors explain a large share of variance, the standard error suggests that unmeasured factors such as informal dumping practices, specific industrial discharges, or variations in waste-collection efficiency would also play important roles. In addition, the use of optical microscopy restricts analysis to particle counts, without identifying polymer types or sources.

Future research should broaden the scope to multiple river systems, apply advanced spectroscopic techniques such as FTIR or Raman spectroscopy to identify polymer types, and integrate socio-economic surveys and land-use mapping to better quantify behavioral and infrastructure drivers. Long-term monitoring is also necessary to assess seasonal and interannual variability, as well as ecological impacts on aquatic biota and to assess the effectiveness of mitigation measures guided by this study.

## Methods

**Fig. 5:**
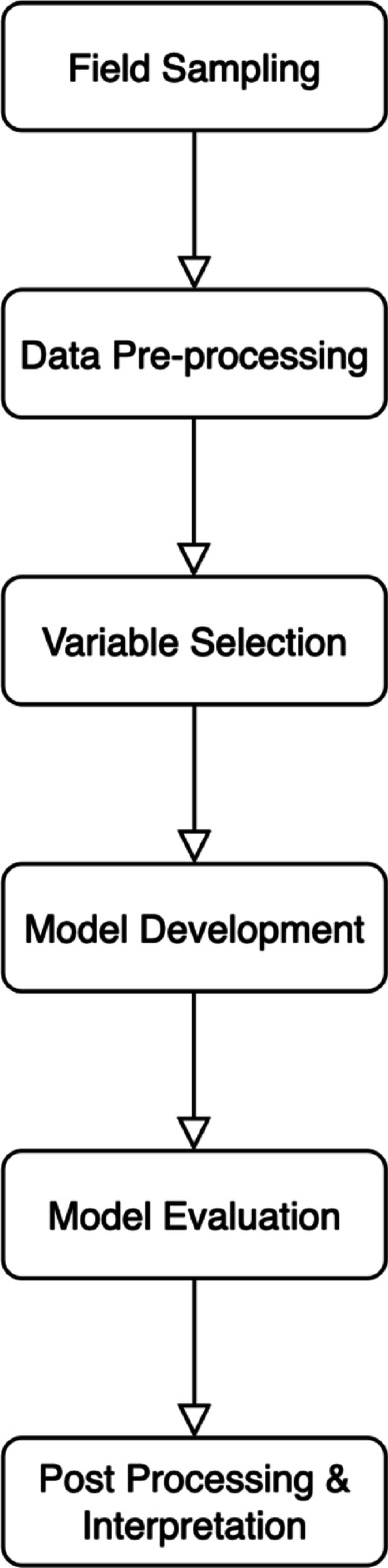
Schematic diagram of the methodological workflow for predicting microplastic concentrations in the Lupit River.

This section outlines each stage of the study shown in Fig. 5. The following schematic diagram provides a clear overview of the step-wise methodological approach used to develop a predictive model of microplastic concentrations in the Lupit River. The process begins with field data collection across four land-use zones during both dry and wet seasons, where microplastic concentrations, macroplastic frequency, and hydrological parameters (width, depth, velocity) were measured. In the pre-processing stage, volumetric flow rate was calculated from field measurements, and a Pearson correlation matrix was used to assess multicollinearity. Variables showing strong correlation were excluded to ensure model stability. Multiple linear regression modeling was performed using JMP Statistical Discovery software which executed statistical analysis, variable significance testing, and model performance evaluation. The model’s output was a predictive equation for estimating microplastic concentrations based on the selected inputs. Results were interpreted in the context of anthropogenic activity, hydrological behavior and seasonal dynamics.

### Field sampling

**Fig. 6:**
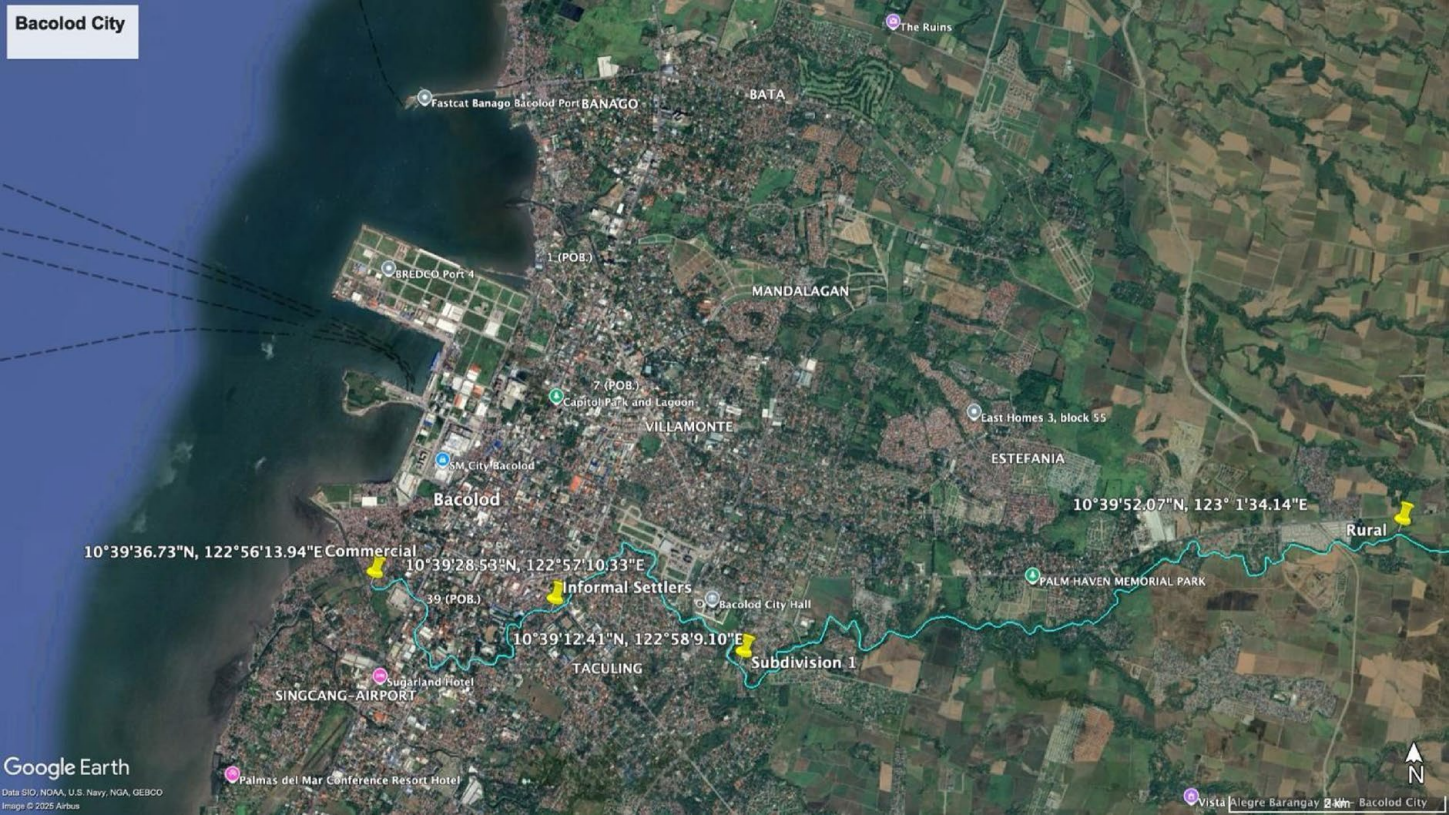
Specific locations of hotspots for each cluster area. This map was generated using Google Earth Pro version 7.3.6.10201^31^. The base satellite imagery is provided by Airbus (© 2025).

Bacolod City is the capital of the Negros Island Region, which is located in the Western Visayas of the Philippines. The Lupit River is one of five major river systems in Bacolod City and is bounded by six barangays (the smallest administrative units in the Philippines, comparable to neighborhoods), which embrace almost 30% of the city’s total population. The river extends to a length of 16.625 km, with 38% of that inhabited by informal settlers who live along the riverbanks roughly halfway to the estuary area. In these areas, the river is highly polluted with solid waste comprising mostly plastics as shown in Fig. 6.

Researchers visually surveyed the river to identify multiple hotspots with significant plastic waste leakage. These hotspots were characterized by the regular presence of visible plastic debris, a lack of formal solid waste collection, and the existence of illegal dumpsites—indicating that waste was not being brought to designated landfill areas for proper disposal^18,19^. As there had been no prior plastic pollution studies in Bacolod City, the most prominent of these hotspots was selected as a baseline site for river sampling. The hotspots within each sector were the sampling sites considered in the study. Figure 6 presents a map of the study area, including the Lupit River and the barangays (local administrative units) it traverses. The figure also highlights the geographical coordinates of the sampling sites for the different sectors. The river was divided into four sectors for sampling: rural, residential, informal settlements, and commercial zone. The river’s outlet into the sea is located near the commercial sector. Specifically, Barangay Granada and Barangay Vista Alegre border the rural segment of the river (1.565 km), Barangay Villamonte borders the residential segment (8.742 km), Barangay 40 and Barangay Vista Alegre border the informal settlement area (1.565 km), and Barangay Singcang borders the commercial segment (3.211 km). The specific GPS coordinates of each identified hotspot were recorded using a GPS tracker.

While the comparison of Lupit River microplastic data to other rivers in the Philippines provides valuable regional context, broadening the scope to include international freshwater systems can enhance the study’s global relevance. Reference^32^) highlight differences in microplastic pollution between developed and developing countries as well as between urban and rural areas, noting the significant impact of human activity. Their study also employed regression models to analyze factors affecting microplastic pollution distribution, similar to the approach used in the present research, demonstrating the broader applicability of this methodological technique in the field of microplastic pollution studies.

### Data pre-processing

This section discusses the methods of collecting the samples and field measurements that were pre-processed to ensure accurate and consistent data across sites and seasons.

The study covered the two seasons in Bacolod City: the wet season from June through November 2023 and the dry season from December 2023 through May 2024. The first sampling was done in November 2023 to capture the conditions during the wet season, with a subsequent sampling in February 2024 to reflect conditions in the dry season.

The river’s physical characteristics were quantified by measuring its depth, width, and flow velocity. Areas of strong flow were visually identified, focusing on the center of the channel and sections near curves or bends, where riverbed morphology influences water velocity. The river’s width between the banks was measured using a range finder. To determine the mean depth, the river cross-section was divided into three equal segments. At each segment, depth measurements were taken using a measuring pole, while the wetted width was recorded with a measuring tape. This procedure was repeated over five trials, and the average depth was calculated accordingly.

**Table 2:** Parameters measured in the lupit river study, with units, ranges, and sectoral means (wet and dry seasons).

Parameter	Unit	Wet season range	Dry season range	Notes / sectoral highlights
River width	m	3.244–17.583	3.244–17.583	Constant per sector; narrowest in rural, widest in commercial
Mean depth	m	0.254–6.116	0.222–2.421	Deepest in rural during wet season (6.116 m); residential consistently shallow (< 0.3 m)
Mean velocity	m/s	0.251–0.521	0.050–0.406	Highest in rural wet (0.521 m/s); lowest in informal settlement dry (0.050 m/s)
Population (per sector)	persons	33,372–73,947	33,372–73,947	Rural largest (73,947); commercial smallest (33,372)
Macroplastic count	items/m·hr	75–177	213–2,011	Large increase in commercial sector during dry season
Microplastic Abundance	particles/m³	1,111–1,778	1,778–2,444	Dry season consistently higher; residential = hotspot

The Lupit River exhibited strong seasonal contrasts in its physical characteristics and pollution levels. River depth and velocity were highest during the wet season (up to 6.12 m and 0.52 m/s, respectively) but declined sharply in the dry season, particularly in the informal settlement sector. Sectoral comparisons showed that the commercial zone was consistently the widest, the residential area the shallowest, and the rural area the deepest and fastest flowing during the wet season. Macroplastic and microplastic loads were consistently greater in the dry season, with the commercial and residential sectors emerging as hotspots, while the rural sector, despite having the largest population, recorded the lowest contamination. These patterns highlight the combined influence of hydrology and land use on plastic pollution dynamics.

The mean river flow velocity was measured using the float method adapted from the hydrological training material^33^ The float method is a widely used and cost-effective approach in field hydrology for estimating river flow velocity when advanced instrumentation is not available^34^. At each sampling site, a 10 foot (3.05 m) straight section of the river was selected, and a half-filled water bottle was released from the upstream to the downstream marker^34^. To maintain consistent measurement, the same type of floating material was used across all sampling locations. The time taken for the float to travel this distance was recorded using a stopwatch and repeated five times to minimize error. Due to the sudden rise in river water levels during fieldwork, only three repetitions were conducted at the commercial area near the tidal zone which posed a safety and logistical challenge. Nevertheless, the consistency of measurements across all sites and the use of multiple trials ensured a low variation between recorded times and their mean was within the range observed across the other sites.

In field hydrology, surface velocity measured using floating objects tends to overestimate the actual mean velocity of the river channel due to minimal friction at the surface. To correct this, a multiplication factor of 0.85 is commonly applied to convert surface velocity to mean velocity. The average time was used to compute the mean channel velocity using Eq. (2):

2 $$\in \:mean\:velocity\:\left( {\frac{{ft}}{s}} \right) = \:\frac{{distance\:\left( m \right)}}{{average\:time\:\left( s \right)}} \times \:0.85\:$$

The volumetric flowrate was computed using the data of the width and average depth using the continuity equation:

3 $$\:volumetric\:flowrate = width\: \times \:average\:depth\: \times mean\:velocity$$

The effect of human activities on river plastic pollution was assessed through targeted cleanups conducted along 100-foot (approximately 31.5 m) sections of the river within each of the four sectors: rural, residential, informal settlements, and commercial zone. In each sector, one section was deliberately selected to standardize the cleanup process across locations and ensure the effort remained manageable. While rapid-survey procedures in large river systems (over 200 km) typically employ 100-meter transects, this methodology was scaled down to fit the smaller Lupit River, which spans less than 17 km. Floating plastic debris was collected and recorded by counting each item to determine the number of items removed from the river per hour. Macroplastic items included packaging materials (e.g., plastic bags, food wrappers, bottles), household items (e.g., shampoo bottles, plastic plates and cutlery, toys), personal care products (e.g., wipes, diapers, napkins, cotton swabs), construction and industrial plastics (e.g., PVC pipes, tarpaulins), and miscellaneous items (e.g., pens, labels, footwear, synthetic textiles).

### Microplastic sampling and analysis

Microplastic was collected by grab sampling. Water samples were taken from the surface of the river, specifically in areas with the highest current velocity. The use of grab sampling over automated water samplers was a strategic choice for the study in Lupit River. This method involves collecting discrete samples, which is highly effective for capturing solid particles like micro- and macroplastics that automated samplers, designed for dissolved substances, might miss. From a practical standpoint, grab sampling is a cost-effective and logistically simple method, requiring no complex equipment, power, or extensive maintenance. This made it ideal for sampling at multiple, difficult-to-access locations within the study area. While it provides a snapshot in time, the data collected from multiple, strategic hotspots offers a reliable and focused assessment of plastic pollution levels, avoiding potential issues like clogging that automated systems might face in a river with significant solid waste. The sampling container was rinsed three times with river water, and the rinse water was discarded downstream from the collection site. The sample container was lowered into the water with the opening facing downward and held to a depth of at least 4 inches below the surface. Three 1.5 L water samples were collected for microplastics analysis.

The sample water’s microplastic content was determined using optical microscopy. Microplastics in water range in size from 5 mm to 0.3 mm^35^. The sample water was filtered through stacked stainless steel mesh sieves with openings of 5.6 mm and 0.33 mm. The sample was rinsed with distilled water to transfer all residual solids to the sieves while also removing salts that may have been present in the sample. To isolate only microplastics in the sample, organic materials were removed by adding 20 mL of 0.05 M aqueous Fe(II) solution to the beaker containing the 0.3 mm size fraction of collected solids^36^. Then, 20 mL of 30% hydrogen peroxide was added to the mixture. The mixture was allowed to sit at room temperature for five minutes and was placed on a hotplate before proceeding. The beaker was removed from the hotplate when gas bubbles were observed on the surface and placed in a fume hood until the boiling stopped. Distilled water was added to slow the reaction in cases in which the mixture overflowed. The mixture was heated to 75 °C for an additional 30 min. If any natural organic material remained visible, 20 mL of 30% hydrogen peroxide was added and the procedure was repeated until no organic material was detectable. The density of the solution was increased by adding approximately 6 g of NaCl per 20 mL of sample to create a ~ 5 M NaCl solution, which was heated to 75 °C until the salt dissolved. The beaker was covered with a watch glass and finally heated to 75 °C on a hotplate. The solution was allowed to cool and was screened using the 0.3 mm–opening sieve. The identifiable microplastics from the 0.3 mm sieve were transferred using forceps to a clean, dry 4 ml vial. The microplastics sample was divided into four parts, and each part was analyzed using a microscope at 40× magnification. The count for each ml of the sample was determined, and the mean of the three replicates from each sampling location was calculated.

### Variable selection

Predictor variables for the multiple linear regression model were selected based on their potential influence on microplastic concentrations, including population density, seasonality, macroplastic abundance and river flow rate. To ensure model reliability and prevent multicollinearity, pairwise correlations among candidate variables were assessed and highly correlated variables were removed. This method allowed the model to retain the most informative and independent predictors, consistent with approaches used in similar studies^32^.

### Model development

This study employed multiple linear regression to model the influence of population, seasonality, macroplastic abundance, and flow rate on microplastic concentrations in the Lupit River. Multiple linear regression analysis was done using JMP Pro (Version 18.0.2^37^ to determine whether the microplastic abundance (MPA) could be predicted by the variables measured during the study. The MPA was modelled in terms of the volumetric flow rate, velocity, width, macroplastic frequency, and population as predictor variables. The analysis was done simultaneously for both the dry and wet seasons. In the statistical model for microplastic abundance, seasonality was included as a categorical predictor variable using a nominal encoding scheme in JMP Pro. The wet season was represented by the value 1, and the dry season by 2. These values served as identifiers for the two distinct environmental conditions rather than as a continuous scale. This approach was justified because it allows the model to directly compare the mean microplastic abundance between the two seasons. The software’s automatic dummy coding handles this categorical variable, creating a binary variable that accurately reflects the fundamental differences in river flow and plastic run-off between the wet and dry periods. This method ensures that the model correctly accounts for the influence of seasonality on microplastic abundance. The Fit Model module in JMP Pro was used for the multilinear analysis. The MPA was specified as the Y, Response variable, and the volumetric flow rate, velocity, width, macroplastic frequency, and population were specified as the Model Effects or predictor variables. The personality used for the analysis was the standard least squares method, and the emphasis was set to “Effect leverage.”

### Model evaluation

Model performance was assessed using JMP Pro outputs. The proportion of data variation explained by the model was assessed using the R² value from the Summary of Fit output, where values between 0.6 and 0.7 are generally considered strong and acceptable for environmental science applications. The *p*-value was reported for the whole model as well as for the individual predictor variables as to whether they had a statistically significant relationship with MPA. Individual leverage plots of the predictor variables were generated with respect to the MPA estimation to determine whether there were points that may have exerted influence on the hypothesis, to spot any unusual patterns or violations of the model assumptions, and to identify any multicollinearity problems. To determine multicollinearity in the regression analysis, the independent variable (MPA) correlation matrix was checked for any strong pairwise correlations. The variance inflation factor (VIF) was computed for each predictor to quantify how much the variance of its estimated coefficient was inflated by correlations with other predictors. A very strong correlation falls between 0.80 and 1.0, and a weak correlation is below 0.39. Any VIF above 5 or 10 for that collinear variable was removed to eliminate redundancy and improve the model’s accuracy and interpretability. The prediction expression was generated by JMP Pro to display the equation used to predict the response^38^.

### Post processing and interpretation

After developing and evaluating the regression model, the outputs were processed to facilitate analysis. Predicted microplastic concentrations were calculated and the residuals examined for patterns or outliers. The results were summarized by season and river sector to highlight spatial and temporal variations.

Model interpretation involved assessing the magnitude and direction of each predictor’s effect on microplastic abundance through regression coefficients. Statistical significance was determined using p-values. Differences between seasons and river sectors were assessed and leverage and influence points were investigated to determine which data points may extremely affect the model in order to provide reliable insights into the distribution of microplastics.

## Data Availability

The datasets generated and analyzed during the current study are not publicly available as the authors lacked permission to share the data but are available from the corresponding author on reasonable request.

## References

[1] Mishra, A., Viswanathan, P. M., Ramasamy, N., Panchatcharam, S. & Sabarathinam, C. Spatiotemporal distribution of microplastics in Miri coastal area, NW borneo: inference from a periodical observation. *Environ. Sci. Pollut. Res.* **30** (46), 103225–103243. 10.1007/s11356-023-29582-7 (2023). 10.1007/s11356-023-29582-7 PMC10567912 37688695

[2] Reineccius, J., Schönke, M. & Waniek, J. Abiotic long-term simulation of microplastic weathering pathways under different aqueous conditions. *Environ. Sci. Technol.* **57** (2), 963–975. 10.1021/acs.est.2c05746 (2023). 36584307 10.1021/acs.est.2c05746

[3] Jain, R. et al. Microplastic pollution: Understanding microbial degradation and strategies for pollutant reduction. *Sci. Total Environ.* **905**, 167098. 10.1016/j.scitotenv.2023.167098 (2023). 37717754 10.1016/j.scitotenv.2023.167098

[4] Roy, P., Mohanty, A. K. & Misra, M. Microplastics in ecosystems: their implications and mitigation pathways. *Environ. Sci. Adv.* **1** (1), 9–29. 10.1039/d1va00012h (2022).

[5] Tirkey, A. & Upadhyay, L. S. B. Microplastics: an overview on separation, identification and characterization of microplastics. *Mar. Pollut. Bull.* **170**, 112604. 10.1016/j.marpolbul.2021.112604 (2021). 34146857 10.1016/j.marpolbul.2021.112604

[6] Pal, D. et al. Microplastics in aquatic systems: A comprehensive review of its distribution, environmental interactions, and health risks. *Environ. Sci. Pollut. Res.* **32**, 56–88. 10.1007/s11356-024-35741-1 (2025). 10.1007/s11356-024-35741-1 PMC11717821 39668270

[7] Yang, W. et al. Impacts of microplastics on immunity. *Front. Toxicol.* **4**, 956885. 10.3389/ftox.2022.956885 (2022). 36238600 10.3389/ftox.2022.956885 PMC9552327

[8] Chen, H., Gibbins, C. N., Selvam, S. B. & Ting, K. N. ). Spatio-temporal variation of microplastic along a rural to urban transition in a tropical river. *Environ. Pollut.* **289**, 117895. 10.1016/j.envpol.2021.117895 (2021). 34364115 10.1016/j.envpol.2021.117895

[9] Hassan, I., Sethupathi, S., J.K. Bashir, M., L. Ahmad, A. & Parthasarathy, P. Tracking microplastics at the source: a comparative study of fluorescent and FTIR microscopy at a drinking water intake in the Perak River, Malaysia. *Environ. Monit. Assess.* **197** (6), Article702 (2025). 10.1007/s10661-025-14127-x PMC12125096 40447941

[10] Li, W. et al. Effects of environmental and anthropogenic factors on the distribution and abundance of microplastics in freshwater ecosystems. *Sci. Total Environ.* **856**, 159030. 10.1016/j.scitotenv.2022.159030 (2023). 36167125 10.1016/j.scitotenv.2022.159030

[11] Guo, M., Noori, R. & Abolfathi, S. Microplastics in freshwater systems: dynamic behaviour and transport processes. *Resour. Conserv. Recycl.* **205**, 107578. 10.1016/j.resconrec.2024.107578 (2024).

[12] Guo, M., A Bon, S. & Abolfathi, S. Undefined. *Water Res.* **282**, 123534. 10.1016/j.watres.2025.123534 (2025). 40262433 10.1016/j.watres.2025.123534

[13] Stride, B., Abolfathi, S., Bending, G. D. & Pearson, J. Hyporheic exchange processes of pore-scale microplastics. *Sci. Total Environ.* **982**, 179573. 10.1016/j.scitotenv.2025.179573 (2025). 40373686 10.1016/j.scitotenv.2025.179573

[14] Stanton, T., Johnson, M., Nathanail, P., MacNaughtan, W. & Gomes, R. L. Freshwater microplastic concentrations vary through both space and time. *Environ. Pollut.* **263**, 114481. 10.1016/j.envpol.2020.114481 (2020). 32276130 10.1016/j.envpol.2020.114481

[15] Talbot, R. & Chang, H. Microplastics in freshwater: A global review of factors affecting Spatial and Temporal variations. *Environ. Pollut.* **292**, 118393. 10.1016/j.envpol.2021.118393 (2022). 34678395 10.1016/j.envpol.2021.118393

[16] Gonzalez-Saldias, F., Sabater, F. & Gomà, J. Microplastic distribution and their abundance along rivers are determined by land uses and sediment granulometry. *Sci. Total Environ.* **933**, 173165. 10.1016/j.scitotenv.2024.173165 (2024). 38740195 10.1016/j.scitotenv.2024.173165

[17] Stride, B., Abolfathi, S., Bending, G. & Pearson, J. Quantifying microplastic dispersion due to density effects. 10.2139/ssrn.4563526 (2023). 10.1016/j.jhazmat.2024.133440 38246058

[18] Tasseron, P. et al. Defining plastic pollution hotspots. *Sci. Total Environ.* **934**, 173294. 10.1016/j.scitotenv.2024.173294 (2024). 38763189 10.1016/j.scitotenv.2024.173294

[19] Maitra, S. P. & Jain, A. Plastic leakage assessment toolkit. *National Institute of Urban Affairs, Climate Centre for Cities*. https://niua.in/c-cube/sites/default/files/Purple%20WMM1%20-%20Plastic%20Waste_2.pdf (2022).

[20] Department of Social Welfare and Development. *DROMIC Report #2 on the effects of El Niño as of 09 March 2024, 6AM*. (2024). https://dromic.dswd.gov.ph/wp-content/uploads/2024/02/DSWD-DROMIC-Report-2-on-the-Effects-of-El-Nino-as-of-09-March-2024-6AM.pdf

[21] Talavera, A. L., Dalida, L. F. M. & Diola, M. B. L. D. Riverine macroplastic survey along the segments of Tullahan river in metro Manila, Philippines. *Front. Environ. Sci.* **12**10.3389/fenvs.2024.1396525 (2024).

[22] Osorio, E. D., Tanchuling, M. A. N. & Diola, M. B. L. D. Microplastics occurrence in surface waters and sediments in five river mouths of Manila Bay. *Front. Environ. Sci.* **9**, 719274. 10.3389/fenvs.2021.719274 (2021).

[23] Espiritu, E. Q. et al. Assessment of quantity and quality of microplastics in the sediments, waters, oysters, and selected fish species in key sites along the Bombong estuary and the coastal waters of Ticalan in San Juan, Batangas. *Philippine J. Sci.* **148** (4), 789–801 (2019).

[24] Argota, H. L., Bajado, J. A., Diola, M. D. & Tanchuling, M. A. N. Macro- and microplastic pollution in Tunasan River, Metro Manila, Philippines. In *Proceedings of the 4th Symposium of the Asian Regional Branch of International Waste Working Group (IWWG-ARB) 2019*. (2018).

[25] Lumongsod, S. L. R. & Tanchuling, M. A. N. Microplastic characterization and analysis in three Makati City creeks. In *Proceedings of the 4th Symposium of the International Waste Working Group–Asian Regional Board (IWWG-ARB) 2019*, (2019).

[26] van Emmerik, T. & Schwarz, A. E. Plastic pollution in rivers: A global review. *Sci. Total Environ.* **743**, 140525. 10.1016/j.scitotenv.2020.140525 (2020).

[27] Windsor, F. M. et al. The role of rivers in the transport of microplastics to marine environments. *Sci. Total Environ.* **646**, 1157–1165. 10.1016/j.scitotenv.2018.07.400 (2019).

[28] Hurley, R. R., Woodward, J. C. & Rothwell, J. J. Microplastic contamination of river beds significantly reduced by catchment-wide flooding. *Nat. Geosci.* **11** (4), 251–257. 10.1038/s41561-018-0070-0 (2018).

[29] Liu, Q., Schnoor, J. L. & McBean, E. Microplastics in food: A review of sources, occurrence, and potential health risks. *Environ. Int.* **146**, 106201. 10.1016/j.envint.2020.106201 (2021). 33129000 10.1016/j.envint.2020.106201 PMC7775884

[30] Chamas, A. et al. Degradation of plastics in the environment and its environmental impacts. *Science* **369** (6510), 1513–1516. 10.1126/science.aba3656 (2020).

[31] Google. *Google Earth Pro* (Version 7.3.6.10201) [Computer software], https://www.google.com/earth/. (2022).

[32] Chen, H., Qin, Y., Huang, H. & Xu, W. A regional difference analysis of microplastic pollution in global freshwater bodies based on a regression model. *Water* **12** (7), 1889. 10.3390/w12071889 (2020).

[33] Arizona. Measuring the Flow of a Stream. The Float Method. https://www.youtube.com/watch?v=W1lUdxE5BGU (2018).

[34] Adzhar, N. F. N. et al. Wan Izatul Asma Wan Talaat, W.I., The travel time of floatable litter of different densities influenced by river flow velocity. *Appl. Sci.*, 13(6), 3450. 10.3390/app13063450 (2023).

[35] Masura, J., Baker, J., Foster, G. & Courtney-Herring, C. Laboratory methods for the analysis of microplastics in the marine environment: Recommendations for quantifying synthetic particles in waters and sediments. *NOAA Technical Memorandum NOS-OR&R 48*, https://repository.library.noaa.gov/view/noaa/10296. (2015).

[36] Zobkov, M. B. & Esiukova, E. E. Microplastics in a marine environment: review of methods for sampling, processing, and analyzing microplastics in water, bottom sediments, and coastal deposits. *Oceanology* **58** (1), 137–143. 10.1134/s0001437017060169 (2018).

[37] SAS Institute Inc. *JMP Pro* (Version 18.0.2) [Computer software]. https://www.jmp.com/ (2024).

[38] JMP Statistical Discovery LLC. *JMP 18 Fitting Linear Models* (JMP Statistical Discovery LLC, 2024).

